# microRNA 490-3P enhances the drug-resistance of human ovarian cancer cells

**DOI:** 10.1186/s13048-014-0084-4

**Published:** 2014-08-31

**Authors:** Shuo Chen, Xi Chen, Yin-Ling Xiu, Kai-Xuan Sun, Zhi-Hong Zong, Yang Zhao

**Affiliations:** Department of Gynecology, The First Affiliated Hospital of China Medical University, Shenyang, 110001 P.R. China; Department of Biochemistry and Molecular Biology, College of Basic Medicine, China Medical University, Shenyang, 110001 P.R. China

**Keywords:** Ovarian cancer cells, microRNA 490-3P, Paclitaxel, Drug resistance

## Abstract

**Background:**

MicroRNAs (miRNAs) are non-coding, single-stranded small RNAs that regulate gene expression negatively, which is involved in fundamental cellular processes. In this study, we investigated the role of miR-490-3P in the development of drug resistance in ovarian cancer cells.

**Methods:**

The human ovarian carcinoma cell line A2780 and A2780/Taxol were exposed to paclitaxel in the presence or absence of microRNA 490-3P transfection, after which cell viability were performed by CCK-8 assay. Reverse transcription polymerase chain reaction (RT-PCR) and western blotting were used to assess the mRNA and protein expression levels of GST-π, MDR1 or P-gp.

**Results:**

Our results showed higher miR-490-3P mRNA expression level in A2780/Taxol cells than in A2780 cells (p < 0.05). Following miR-490-3P transfection, both A2780 and A2780/Taxol cells showed decreased sensitivity to paclitaxel. The mRNA expression levels of MDR1, GST-π (*p* < 0.05) and protein expression levels of P-gp, GST-π were down-regulated after miR-490-3P transfection in comparison to mock and negative control cancer cells.

**Conclusion:**

Our results demonstrate for the first time that microRNA 490-3P may be involved in the development of drug resistance in ovarian cancer.

**Electronic supplementary material:**

The online version of this article (doi:10.1186/s13048-014-0084-4) contains supplementary material, which is available to authorized users.

## Introduction

Ovarian cancer remains the most common cause of death from gynecological malignancies [1,2]. Among them, epithelial ovarian cancer (EOC) is the fifth leading cause of cancer death in women and the most lethal gynecologic malignancy in the world [3]. Given that recurrence and metastasis seriously affect the prognosis of ovarian cancer, the five-year survival rate for all stages of ovarian cancer has been estimated to be 35–38% [3,4]. Primary treatment of ovarian cancer is surgical resection of visible disease followed by adjuvant chemotherapy, paclitaxel, for example, is commonly used in the treatment of several types of cancer, including ovarian cancer. Paclitaxel primarily kills cancer cells via microtubule stabilization [5]. However, since numerous patients with ovarian cancer who initially respond to paclitaxel-therapy eventually relapse with a drug-resistant form of the disease, their five-year survival rate was not improved. Thus, it is extremely necessary to identify novel and efficient biomarkers used as therapeutic targets for human EOC, which also become the focus of recent research.

MicroRNAs (miRs) are small non-coding RNA molecules 18-25 nucleotides in length, which negatively regulate gene expression at the post-transcriptional level by binding to imperfect complementary sites in the 3’-UTR (untranslated region) of their target messenger RNA transcripts [6]. Accumulating studies had showed that the discrepancy of microRNA expression may contribute to cell proliferation [7-9] and tumor development [10-14]. Thus microRNAs have been turned out to be promising diagnostic and prognostic molecular biomarkers as well as therapeutic targets in cancers [15,16]. In recent years, more attention has been paid to the role of miRNAs in reversing drug resistance. Studies showed that microRNAs can play an important role in modulating the sensitivity of cancer cells to chemotherapeutic agents [17,18]. However, Zhimin Li et al. reported that MiR-27a may up-regualte MDR1/P-glycoprotein expression by targeting HIPK2 in human ovarian cancer cells [19], Li H et al. also reported that miR-106a may be involved in the development of drug resistance and the regulation of PDCD4 expression [20].

Until now, miR-490-3P has been validated to act as a regulator of cell proliferation, migration, invasion, or in the EMT in hepatocellular carcinoma cells and vascular smooth muscle cells [21,22], but its function in ovarian cancer cell lines has not been reported yet. This is the first study to determine the drug-resistance function of miR-490-3P in the ovarian cancer cell lines.

## Materials and methods

### Reagents

The miRNA 490-3P mimic with the sense strand sequence 5’-CAA CCU GGA GGA CUC CAU GCU G-3’ and the control (designated Mock) sense strand sequence 5’-ACU ACU GAG UGA CAG UAG A-3’ were used in transient transfection of ovarian carcinoma cell lines.

### Cell culture

Ovarian carcinoma cell lines, A2780 (serous cystic adenocarcinoma) and Paclitaxel-resistant A2780 (A2780/Taxol, supplemented with 800 ng mL^−1^ Paclitaxel), were purchased from ATCC. Cells were maintained in RPMI 1640 (A2780/Taxol) and DMEM (A2780) medium supplemented with 10% fetal bovine serum, 100 units mL^−1^ penicillin and 100 μg mL^−1^ streptomycin. Cell lines were kept in a humidified atmosphere of 5% CO_2_ at 37°C with or without paclitaxel treatment and miR-490-3P transfection.

### Cell viability assay

Cell Counting Kit-8 (CCK-8, Japan) was employed to determine the number of viable cells via a colorimetric assay. Briefly, 2.5 × 10^3^ cells/well were seeded to a 96-well plate and allowed to adhere. At different time points, 10 μL of CCK-8 solution was added into each well of the plate and the plate was subsequently incubated for 3 h at 37°C prior to recording of the optical density at 450 nm.

### Real-time reverse transcription polymerase chain reaction (real-time RT-PCR)

Total RNA was extracted from the ovarian carcinoma cell lines using TRIzol® (Takara, Kyoto, Japan). Real-time RT-PCR was performed from 2 μg of total RNA using AMV reverse transcriptase and random primers (Takara, Kyoto, Japan). PCR primers were designed according to the sequences in GenBank and are listed in Additional file 1: Table S1. Amplification of cDNA was performed according to the manufacturer’s protocol using an SYBR Premix Ex Taq II kit (Takara, Kyoto, Japan) and *GAPDH* as an internal control. Briefly, RT-PCR amplification of cDNA for each primer was carried out in a final volume of 20 μL, containing 10 μL SYBR Premix Ex Taq (×2), 0.08 μL primers, 0.4 μL ROX reference dye and 1 μL template cDNA (50 μg μL^−1^). The protocol parameters were as follows: initial incubation at 95°C for 30 s followed by 40 cycles of denaturation at 95°C for 5 s and annealing at 60°C for 34 s. All the PCR experiments were accompanied with a no-template control and *GAPDH* as an internal control. The relative gene expression level (the amount of target normalized to the endogenous control gene) was calculated using the comparative CT method: 2^-ΔΔCt^. The sequences of primers for real-time quantitative PCR are supplied in Additional file 1: Table S1.

### Western blot analysis

Protein assays were performed according to the Bradford method using the Bio-Rad protein assay kit (Bio-Rad, Hercules, CA, USA). Denatured proteins were separated by sodium dodecyl sulfate-polyacrylamide gel electrophoresis (SDS-PAGE) on 12% acrylamide gels, and then transferred to Hybond™ membranes (Amersham, Germany). The membranes were blocked overnight in 5% skimmed milk in Tris-buffered saline with Tween® 20 (TBST; 10 mM Tris-HCl, 150 mM NaCl, 0.1% Tween® 20). For immunoblotting, the membranes were incubated for 1 h with the P-gp (P-glycoprotein, Santa Cruz Biotechnology, Santa Cruz, CA, USA) and GST-π (Abcam, Cambridge, UK) antibody, rinsed with TBST and incubated with anti-goat IgG antibodies conjugated to horseradish peroxidase (HRP; Dako, Carpinteria CA, USA) at a dilution of 1:1000. After applying electrochemiluminescent (ECL)-Plus detection reagents (Santa Cruz Biotechnology, Santa Cruz, CA, USA), the protein bands were visualized using an X-ray film (Fujifilm, Tokyo, Japan). The immunoblots were washed with western blotting (WB) stripping buffer (pH 2–3; Nacalai, Tokyo, Japan) and probed using a monoclonal antibody specific for GAPDH (1:1000; Proteintech Group, Chicago, USA).

### Statistical analysis

Statistical evaluation was performed using Spearman’s rank correlation coefficient to analyze ranked data, and the Mann–Whitney U test to differentiate the means of different groups. A *p*-value of <0.05 was considered statistically significant. SPSS v. 10.0 software (SPSS, Chicago, IL, USA) was employed to analyze all data.

## Results

### miR-490-3P decreased sensitivity of the A2780 and A2780/Taxol cells to paclitaxel

**Figure 1:**
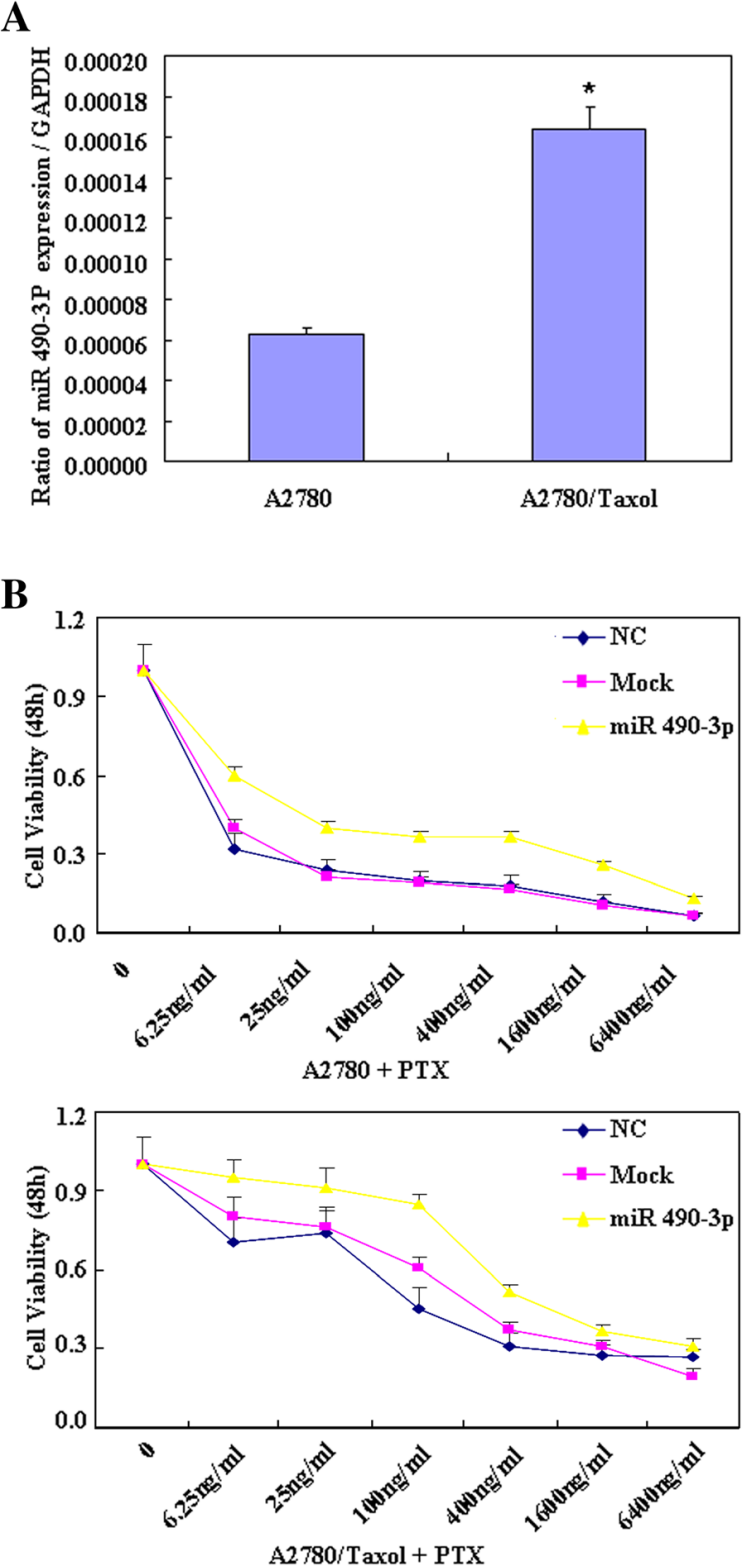
**miR-490-3P decreased sensitivity of the A2780 and A2780/Taxol cells to paclitaxel.** RT-PCR results showed that A2780/Taxol has higher miR-490-3P mRNA expression level than A2780 **(A)**. CCK-8 cell proliferation assays show that after miR-490-3P transfection, the sensitivity of both A2780 and A2780/Taxol cells transfected with miR-490-3P mimics to paclitaxel was decreased compared with that treated with NC or Mock cells **(B)**. Results are representative of three separate experiments; data are expressed as the mean ± standard deviation.

RT-PCR results showed that A2780/Taxol has higher miR-490-3P mRNA expression level than A2780 (Figure 1A, p < 0.05). Figure 1B showed that the sensitivity of A2780 cells transfected with miR-490-3P mimics to paclitaxel was decreased compared with negative control cells or mock transfected cells, the same results was with A2780/Taxol. Besides, A2780/Taxol shows greater resistance to paclitaxel than A2780.

### miR-490-3P down-regulate expression of MDR1/P-gp and GST-π

**Figure 2:**
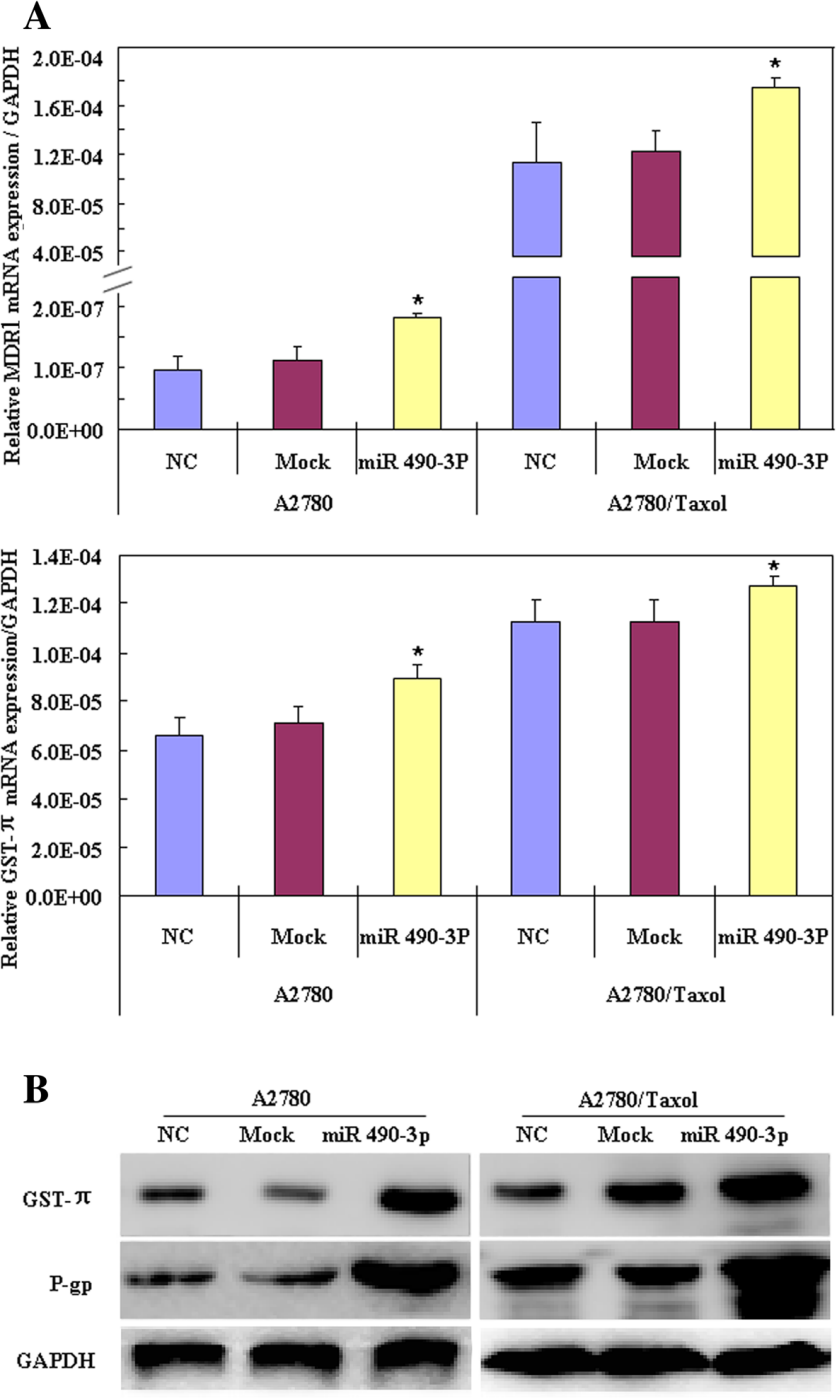
**miR-490-3P down-regulate MDR1/P-gp and GST-π expression.** RT-PCR results showed that after microRNA 490-3P transfection, the mRNA expression levels of MDR1 and GST-π in A2780 and A2780/Taxol cell lines were higher than negative control cells or mock cells (**A**, p < 0.05). Western blot analysis showed that the protein expression levels of P-gp and GST-π were also higher than those of negative control cells and mock cells (**B**, p < 0.05).

RT-PCR results showed that after transfected with mimics of microRNA 490-3P, the mRNA expression levels of MDR1 and GST-π in A2780 and A2780/Taxol cell lines were higher than those observed in negative control cells or mock transfected cells (Figure 2A, *p < 0.05*). At the same time, western blot analysis of the protein expression levels of P-gp and GST-π were also higher than those of negative control cells and mock transfected cells (Figure 2B).

## Discussion

Accumulating evidence nowadays revealed that aberrant microRNA expression is strongly implicated in the development of drug resistance. They may affect the expression of target proteins which could be drug transporters, drug targets or cell apoptosis and cell-cycle-related components, resulting in variations of sensitivity of cells to chemo-therapeutic drugs. Studies showed that miR-21, 106a, 27a, 487, for example, may be involved in the development of drug resistance through regulating relative gene expression [17-20,23]. The studies about the roles of miRNAs in the development of drug resistance have attracted much attention nowadays.

Studies reveal that miR-490-3P overexpression leads to inhibition of cell proliferation via G1-phase arrest. Gu et al. reported that miR-490-3p inhibits proliferation of A549 lung cancer cells [24], and Zhang et al. showed that miR-490-3p modulates cell growth and epithelial to mesenchymal transition (EMT) of hepatocellular carcinoma cells [21]. These studies suggest us that miR-490-3P may also contribute to tumor development; however, its function in the development of drug resistance hasn’t been studied.

Our RT-PCR results showed that A2780/Taxol has higher miR-490-3P mRNA expression level than A2780. To investigate whether miR-490-3P could modulate the sensitivity to paclitaxel, we transfected A2780/Taxol and A2780 cells with mimics of miR-490-3P respectively, and then the cells were treated with a series of concentrations of paclitaxel. Our results showed that the sensitivity of both A2780 and A2780/Taxol cell lines transfected with miR-490-3P mimics to paclitaxel was decreased compared with negative control or mock cells, suggesting that microRNA 490-3P may be involved in the development of drug resistance in ovarian cancer cells.

Multidrug resistance (MDR) is one of the major reasons chemotherapy-based treatments fail. Of the many mechanisms of MDR, the high expression of the human MDR1 gene and the P-glycoprotein (P-gp) transporter encoded by MDR1 is an important focus of research [25]. Tumor cells that overexpress MDR1/P-gp usually show resistance to various chemotherapeutics [26]. Previous studies on the efflux pump have shown that P-gp plays an important part, as it pumps drug substance outside to reduce cytotoxicity presented by cancer cells and enhances the resistance of carcinoma to chemotherapeutics. The drug resistance presented by cancer cells can be effectively induced by up-regulating P-gp expression and function [27-29]. Besides, GST-π (the π isoform of protein enzyme - glutathione S-transferase), one of the members of the glutathione S-transferase (GST) family, is responsible for excessive intensity of detoxification of cytostatics, and have been shown to have functional polymorphisms that may affect drug metabolism and influence the effects of chemotherapy and survival from cancer [30]. Beeghly A et al. showed that reduced GST function may improve ovarian cancer survival after post-operative chemotherapy; evaluation of GST functional polymorphisms may help to predict ovarian cancer prognosis [31].

Our RT-PCR results showed that after transfected with mimics of microRNA 490-3P, the mRNA expression levels of MDR1 and GST-π in A2780 and A2780/Taxol cell lines were higher than those observed in negative control cells or mock transfected cells. At the same time, western blot analysis showed that the protein expression levels of P-gp and GST-π were also higher than those of negative control cells and mock transfected cells, thus, we suggest that microRNA 490-3P may be involved in the development of drug resistance through regulating MDR1/P-gp and GST-π expression in ovarian cancer cells. Above all, we suggest that when microRNA 490-3P was used to reduce ovarian carcinoma’s recurrence, invasion and metastasis, it’s better not to choose paclitaxel for chemotherapy, and we must pay attention to the impact of microRNA 490-3P when using chemotherapy drugs for clinical treatment, drug trials must be conducted.

In conclusion, we demonstrated for the first time that microRNA 490-3P may be involved in the development of drug resistance in ovarian cancer cells. The aberrant specific molecular mechanisms need further study and its clinical manipulation also needs to be cautiously considered in future work.

## Conclusions

We demonstrated for the first time that miR-490-3P may be involved in the development of drug resistance in ovarian cancer cells. The aberrant specific molecular mechanisms need further study and its clinical manipulation also needs to be cautiously considered in future work.

## Additional file

Additional file 1: Table S1. Primers for RT-PCR.
