# Erratum to: microRNA 490-3P enhances the drug-resistance of human ovarian cancer cells

**DOI:** 10.1186/s13048-015-0194-7

**Published:** 2015-12-01

**Authors:** Shuo Chen, Xi Chen, Yin-Ling Xiu, Kai-Xuan Sun, Zhi-Hong Zong, Yang Zhao

**Affiliations:** Department of Gynecology, The First Affiliated Hospital of China Medical University, Shenyang, 110001 P.R. China; Department of Biochemistry and Molecular Biology, College of Basic Medicine, China Medical University, Shenyang, 110001 P.R. China

## Erratum

After publication of [1] it came to the authors’ attention that the word “up-regulate” was revised incorrectly into “down-regulate”. In Page 1, Abstract and Results section: the last sentence “The mRNA expression levels of MDR1, GST-π....were down-regulated after miR-490-3P transfection…” should be “…were up-regulated…” In Page 3, Results: the title of the second paragraph was “miR-490-3P down-regulate expression of MDR1/P-gp and GST-π”, should be “miR-490-3P up-regulate…” In Page 4, the title of Figure 2 Legend should be “miR-490-3P up-regulate expression of MDR1/P-gp and GST-π”.
