# Supplementary material for: microRNA 490-3P enhances the drug-resistance of human ovarian cancer cells
Source: J Ovarian Res. 2014 Aug 31;7:84. doi: 10.1186/s13048-014-0084-4 (PMC4158041; doi:10.1186/s13048-014-0084-4)
Supplement: Additional file 1: Table S1. — Primers for RT-PCR. [file 13048_2014_84_MOESM1_ESM.doc]

**Additional file 1: Table S1:** Primers for RT-PCR

| **Gene** | **Primer sequence** | **Target sequence** | **AT**  **(^o^C)** | **Product size (bp)** | **Extension time (sec)** |
| --- | --- | --- | --- | --- | --- |
| *GST-π* | F: 5'-TTTCGCCGCCGCAGTCT-3'  R: 5'-TCCACGGTCACCACCTCCTC-3' | NM_000852  225-359 | 60 | 135 | 34 |
| *MDR1* | F: 5'-AGGCTATCATTACTCTTTACC-3'  R: 5'-TCTGGCTTCCGTTGC-3' | NM_000927  24-184 | 60 | 161 | 34 |
| *GAPDH* | F: 5’-CAATGACCCCTTCATTGACC-3’  R: 5’- TGGAAGATGGTGATGGGATT-3’ | NM_ 002046.3  201-335 | 60 | 135 | 34 |

AT = annealing temperature
